# Moving Mindfully: The Role of Mindfulness Practice in Physical Activity and Health Behaviours

**DOI:** 10.3390/jfmk6010019

**Published:** 2021-02-10

**Authors:** Dev Roychowdhury

**Affiliations:** Health Research Hub, DR ACADEMY, Melbourne, Victoria 3011, Australia; info@drdevroy.com

**Keywords:** mindfulness, mindful practice, physical activity, health, health behaviour, health outcome, well-being

## Abstract

Participation in regular physical activity yields numerous psychological and physical health benefits. Despite this, a large proportion of the global population is increasingly becoming inactive and sedentary, which has been linked to various causes of morbidity and mortality. One practice that has been found to encourage healthy participation in physical activity and associated health behaviours is mindfulness. Mindfulness practices have been consistently linked to higher levels of physical activity participation. However, the relationship between mindfulness practices and physical activity remains ambiguous. This present paper comments on the role of mindfulness practice in physical activity and health behaviours. Implications for future research and practice have also been addressed.

## 1. Introduction

Research has consistently linked engaging in regular physical activity (PA) with a wide range of positive health outcomes and well-being [1,2]. Despite the evidence and continued public health campaigns to promote PA in the general population [3], recent trends indicate an increase in levels of physical inactivity and sedentarism [2,4,5]. Given physical inactivity and sedentary behaviours have been found to be associated with high levels of global morbidity and mortality, there is an insistent need to motivate people to increase the amount of PA they undertake [6,7,8].

Although studies examining participation motivation in PA have highlighted key reasons people nominate for engaging in different types of PA [6,7,8,9,10,11,12,13,14], suitable interventions are yet to be developed and implemented that would use this knowledge effectively. Research indicates that lifestyle interventions that were intended to increase the levels of PA only resulted in ordinary changes in marked behaviours [15,16,17,18], along with limited long-term outcomes [19,20,21]. Moreover, such interventions were found to have low adherence and high dropout rates in the short [22] and long term [23,24].

Novel interventions are, therefore, required that would not only help understand the mechanisms of successful PA behaviour change but also address the barriers to PA that typically affect PA uptake, maintenance, and adherence. In recent years, mindfulness practices have garnered significant attention in the literature, predominantly because of their salutary health benefits and public acceptance [25,26]. Moreover, mindfulness practices have also received greater emphasis due to their potential to address barriers to change [27]. The purpose of this paper is to briefly describe mindfulness and examine its relationship with PA and health behaviours.

## 2. Mindfulness

During the past two decades, interest in mindfulness practices has skyrocketed in the scientific literature and public domain. Despite this, accurate representation and scientific examination of mindfulness and its correlates have been ambiguous and indefinite. Mindfulness, stripped of its original foundational orientation, has now been commodified and repurposed as a magical panacea for different types of human conditions and ailments, which has muddled the understanding of mindfulness practices and created methodological issues in determining their purpose and potential psychophysiological benefits (e.g., [28,29]).

Mindfulness emerged as a meditative practice thousands of years ago in the Vedic traditions of Hinduism in ancient India. It forms a critical element in Eastern traditional and spiritual philosophies and is cultivated through rigorous training [25,26,28]. Yet, acceptance of mindfulness practices has only recently entered the parlance in the Western context. Mindfulness, in the West, has been simply characterized as the ability to pay moment-to-moment and non-judgmental attention. Roychowdhury [25] argues that although it would be nearly impossible to encapsulate the essence of mindfulness, there are three fundamental concepts that lie at its core:

(a) Present-moment awareness: an individual’s ability to focus attention in the here and now
(b) Equanimity: an individual’s ability to maintain a sense of balance between helpful and unhelpful thoughts, emotions, and/or sensations
(c) Non-doing: an individual’s ability to allow life to unfold extemporaneously

## 3. Mindfulness, Physical Activity, and Health Outcomes

There is a substantial amount of research linking the efficaciousness of mindfulness to a range of physical and psychological health outcomes. Studies examining the associations among mindfulness, PA, and health behaviours have concluded that higher levels of mindfulness lead to higher levels of PA [30,31,32], self-efficacy [24], positive emotions [33,34], healthy eating [31,35], better quality of sleep [30,31,35,36], satisfaction [37], and well-being [38]. Conversely, it was also found that individuals who were successful at maintaining PA tended to score higher on measures of mindfulness [39] and that participation in a single session of mindful PA was shown to positively alter individuals’ mood [40]. Furthermore, Zvolensky and colleagues [41] examined the role of mindfulness-based attention in predicting perceived health status and functioning in a community sample of young adults and found that greater levels of mindfulness-based attention were associated with perceptions of better physical and psychological functioning.

Research on mindfulness practices have indicated that engaging in mindfulness-based behaviours increases adaptive and inhibits maladaptive health behaviours. For instance, it has been found that individuals who scored higher on mindfulness measures reported lower levels of substance use, smoking, binge eating, and risky sexual behaviours (e.g., [30,35,42,43,44]). It is, therefore, evident that mindfulness practices facilitate involvement in positive and healthy behaviours whilst simultaneously inhibiting participation in negative or unhealthy behaviours.

## 4. Discussion

The profoundly positive effect of mindfulness on a range of PA and health behaviours is widely documented in the literature (e.g., [30,31,32,33,34,35,36,37,38,39]). It is evident that individuals with higher scores on mindfulness measures tend to engage in adaptive and healthy behaviours and report better physical and psychological functioning (e.g., [30,31,32]). Despite the evidence, the mechanisms underlying mindfulness practices and how they enable engagement in PA and health behaviours are relatively unclear.

The perplexity surrounding the semantics and conceptualization of mindfulness in research and practice renders it arduous to define, observe, and measure. Additionally, as research on mindfulness has increasingly saturated the scientific literature and public discourse, the characterization and subsequent utilization of mindfulness has attracted warranted debate and criticisms (e.g., [26,28]). For instance, mindfulness practices have often been misattributed to Buddhism, despite evidence that these practices originated in Hindu philosophy that pre-date Buddhist teachings by thousands of years [28]. Furthermore, it has been argued that the commodification of contemporary mindfulness as a magical panacea not only serves capitalistic endeavours but may also prove detrimental for individuals with certain psycho-medical conditions and/or who do not have proper training in such practices [26,28,29]. Considering the description and classification of mindfulness in the literature coupled with public discussion has largely been unsystematic and unidimensional, future research should focus on developing a comprehensive and holistic understanding of mindfulness, both in theory and practice.

Future research should investigate how mindfulness mechanisms enable and maintain PA participation and healthy behavioural choices. For instance, the cultivation of present-moment awareness may enable individuals to focus on relevant PA tasks in the present moment. Moreover, the process of equanimity may allow individuals to choose and maintain healthy PA behaviours without being swayed by unhelpful or unwanted thoughts, distractions, and/or sensations. Finally, practicing non-doing perhaps trains individuals to allow their growth to happen in due course, without having to rush their participation or progress in PA. It is likely that these core mindfulness processes (of present-moment awareness, equanimity, and non-doing) foster a sense of attention, acceptance, and calmness that creates an open and healthy orientation and attitude towards PA whilst simultaneously inhibiting unhelpful and unwanted distractions and habitual tendencies, thereby creating a healthy sense of agency and self-control. A state of mindfulness may assist individuals to focus on their PA by becoming more attuned to the activity at hand, observing their thoughts, and being less reactive to unwanted or unhelpful thoughts or emotions. This may be especially pertinent when engaging in or maintaining PA becomes particularly arduous (e.g., uncomfortable sensations or pain, task/PA complexity, goal-ambiguity, and judgement of self or others). Thus, it is plausible that mindfulness practices succor individuals to cultivate self-monitoring, self-control, and persistence skills. Therefore, increased mindfulness training and heightened awareness are likely to support self-regulatory mechanisms that are ultimately responsible for PA behaviours. Future research and practice in this domain should examine the mechanisms underlying mindfulness practices and how they promote healthy PA behaviours and inhibit unhealthy or risky actions.

It is evident from the literature that higher levels of mindfulness promote healthy engagement in PA (e.g., [30,31,32]) and that individuals who are involved in regular forms of PA tend to score higher on measures of mindfulness (e.g., [39]). Despite the symbiotic relationship between mindfulness and PA, the direction of this relationship still remains unclear. It is plausible that more mindful individuals—due to their open, aware, and accepting orientation—tend to prioritize healthy behaviours (such as PA involvement) over others. Likewise, it is conceivable that engaging in healthy behaviours (such as regular PA), which yield salutary psychophysiological benefits, tend to make individuals more mindful about their choices–consequences outcome (i.e., choosing to consciously participate in PA to obtain health benefits). Considering the health benefits and reciprocity of mindfulness practices and PA behaviours, more research is warranted in this domain.

Mindfulness practices and healthy PA behaviours are both cultivated through disciplined training. That is, with precise preparation and sustained efforts, individuals move from a novice state to a more grounded and experienced form. It must be noted that such mindful efforts and PA behaviours take time to cultivate and are affected by myriad internal and external dynamics. Therefore, understanding the temporal, contextual, and existential dimensions in which such behaviours subsist is crucial to understanding the relationship between mindfulness practices and healthy PA behaviours and how they affect the behaviour change process.

Future research and practice in this context should examine mindfulness practices across a wide range of populations, cultures, and behaviours to ascertain the mechanisms and processes that characterize participation in different types of PA. Similarly, the relationship between people who tend to engage in regular PA and their form of mindfulness must also be explored to determine how healthy behaviours promote a state of mindfulness in individuals. These have huge implications for the global population that is increasingly becoming physically inactive and sedentary. Understanding what makes individuals more mindful and encourages them to engage in different types of PA will ultimately aid health researchers and practitioners to develop and implement targeted campaigns to increase population mindfulness and the amount of PA people undertake, which will enhance satisfaction and well-being and help tackle a number of lifestyle-related ailments.

## Data Availability

The data that support the findings of this study are available from the corresponding author upon reasonable request.

## Author Biography

Dev Roychowdhury is a researcher, pracademic, and consultant in mindfulness, motivation, mental health, and performance psychology. He is currently serving as the Research Director at DR ACADEMY and Executive Member of Managing Council of the Asian South-Pacific Association of Sport Psychology. He obtained his doctoral degree in psychology in Australia and has extensive lecturing, research, and industry experience. Dev Roychowdhury has lectured at Monash University and Victoria University in Australia and served as a uniformed and commissioned Army Officer in the Australian Defence Force. He takes keen interest in research and is regularly invited to conduct seminars and workshops. For more information, please visit https://www.drdevroy.com.
